# Non-invasive imaging using reporter genes altering cellular water permeability

**DOI:** 10.1038/ncomms13891

**Published:** 2016-12-23

**Authors:** Arnab Mukherjee, Di Wu, Hunter C. Davis, Mikhail G. Shapiro

**Affiliations:** 1 Division of Chemistry and Chemical Engineering, California Institute of Technology, 1200 East California Boulevard, MC 210-41, Pasadena, California 91125, USA; 2 Division of Engineering and Applied Sciences, California Institute of Technology, Pasadena, California 91125, USA

## Abstract

Non-invasive imaging of gene expression in live, optically opaque animals is important for multiple applications, including monitoring of genetic circuits and tracking of cell-based therapeutics. Magnetic resonance imaging (MRI) could enable such monitoring with high spatiotemporal resolution. However, existing MRI reporter genes based on metalloproteins or chemical exchange probes are limited by their reliance on metals or relatively low sensitivity. Here we introduce a new class of MRI reporters based on the human water channel aquaporin 1. We show that aquaporin overexpression produces contrast in diffusion-weighted MRI by increasing tissue water diffusivity without affecting viability. Low aquaporin levels or mixed populations comprising as few as 10% aquaporin-expressing cells are sufficient to produce MRI contrast. We characterize this new contrast mechanism through experiments and simulations, and demonstrate its utility *in vivo* by imaging gene expression in tumours. Our results establish an alternative class of sensitive, metal-free reporter genes for non-invasive imaging.

## Abstract (web summary)

Magnetic resonance imaging combined with molecular reporters can visualise cellular functions in intact organisms. Here Mukherjee *et al*. present a cellular imaging approach based on intracellular changes in water diffusion using human aquaporin 1 gene as a genetically encoded reporter for MRI.

The ability to image gene expression within the context of living mammalian organisms is critical for basic biological studies and the development of cellular and genetic therapeutics. However, most genetically encoded reporters, based on fluorescent and luminescent proteins^1^,^2^,^3^ have limited utility in this context due to the poor penetration of light into deep tissues^4^,^5^. In contrast to optical techniques, magnetic resonance imaging (MRI) enables the acquisition of *in vivo* images with excellent depth penetration and high spatial and temporal resolution. Consequently, there is intense interest in the development of genetically encoded reporters for MRI^6^,^7^,^8^,^9^,^10^,^11^,^12^,^13^,^14^,^15^,^16^,^17^,^18^,^19^,^20^,^21^,^22^,^23^,^24^,^25^,^26^. Previous efforts to develop such reporters have focused primarily on two classes of proteins. In one class, metalloproteins and metal ion transporters are overexpressed to enrich the paramagnetic content of cells, thereby enhancing nuclear relaxation rates and producing contrast in *T*_1_- or *T*_2_-weighted MRI^9^,^12^,^13^,^14^,^15^,^16^,^17^,^18^,^19^,^25^,^26^,^27^. In the second strategy, proteins with large numbers of basic or acidic amino acids are used to generate contrast through chemical exchange saturation transfer (CEST) between protein-bound and aqueous protons^6^,^8^,^21^,^22^,^28^. Each of these pioneering approaches has significant limitations. Metal-based reporters can be hindered by metal ion bioavailability and toxicity^29^,^30^,^31^,^32^,^33^,^34^,^35^, whereas CEST reporters tend to require high expression levels to achieve observable contrast^6^,^21^,^22^. Hence, a major need exists for new MRI reporter genes that do not require metals and can be detected at low levels of expression.

Here we introduce an entirely new class of non-metallic MRI reporter genes that work by modulating water diffusivity across cell membranes. Diffusion-weighted imaging (DWI) is a well-established MRI technique used in applications ranging from basic biophysical studies to the diagnosis of diseases such as stroke^36^,^37^,^38^,^39^,^40^,^41^,^42^,^43^. Diffusion-weighting is commonly achieved by applying a pair of pulsed magnetic field gradients, which dephase nuclear spins in proportion to how far they diffuse in the time interval between the two pulses^41^,^44^,^45^. Accordingly, water molecules that diffuse more freely have more severely dephased proton spins and appear darker in DWI (Fig. 1a). In biological tissues, the effective diffusion coefficient of water depends on several parameters, including its local diffusivity in intracellular and extracellular compartments, the relative volume fraction occupied by cells and the transport of water across the plasma membrane^46^,^47^,^48^,^49^,^50^. Noting the strong influence of the last factor^46^,^51^,^52^, we hypothesized that facilitating the transmembrane diffusion of water by overexpressing water-permeable channels would result in enhanced contrast in DWI.

Towards this end, aquaporins are a highly conserved family of tetrameric integral membrane proteins that mediate the selective exchange of water molecules across the plasma membrane in a wide range of cell types^53^,^54^,^55^,^56^,^57^,^58^. Previously, endogenous aquaporin expression has been correlated with water diffusivity and DWI signals in several disease states^52^,^57^,^58^. However, to the best of our knowledge, aquaporins have not hitherto been described as MRI reporter genes. In this work, we introduce human aquaporin 1 (AQP1) as a new genetically encoded reporter for diffusion-weighted MRI. This reporter requires no metals, is non-toxic in several cell lines and *in vivo* tumours, produces contrast orthogonal to paramagnetic and CEST reporters and is detectable when expressed at low levels and in small subsets of cells. We characterize the imaging performance and mechanisms of AQP1 through live-cell experiments and Monte Carlo models, and demonstrate its utility by imaging tumour gene expression *in vivo*.

## Results

### Aquaporins serve as reporters for diffusion-weighted MRI

**Figure 1:**
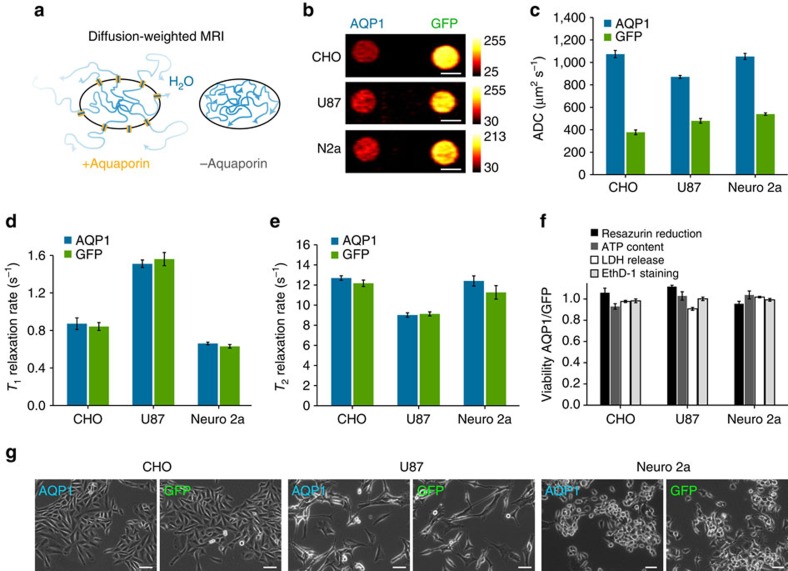
AQP1 functions as a genetically encoded reporter for diffusion-weighted MRI. (**a**) Illustration of the impact of aquaporin expression on water diffusion across the cell membrane and the resulting decrease in diffusion-weighted signal intensity. (**b**) Diffusion-weighted images of CHO, U87 and Neuro 2a cell pellets expressing AQP1 or GFP, acquired using a *b*-value of ∼1,000 s mm^−2^. Scale bars, 3 mm. (**c**) ADC of water in CHO, U87 and Neuro 2a cells expressing AQP1 relative to GFP controls, measured at Δ_eff_=398 ms. Transgene expression in CHO cells was induced with 1 μg ml^−1^ doxycycline, whereas U87 and Neuro 2a cells express AQP1 from a constitutive promoter. *n*=4 (U87, Neuro 2a) and 5 (CHO) biological replicates. (**d**) Longitudinal (*T*_1_) and (**e**) transverse (*T*_2_) relaxation rates in cells expressing AQP1 or GFP. *n*=3 (Neuro 2a, CHO) or 4 (U87) biological replicates. (**f**) Cell viability on AQP1 or GFP expression. *n*=12 (resazurin assay), 6 (ATP content), 4 (LDH release) and 3 (ethidium staining) biological replicates. Error bars±s.e.m. (**g**) Phase-contrast images of CHO, U87 and Neuro 2a cells expressing AQP1 or GFP. Scale bars, 10 μm.

To evaluate AQP1 as a genetically encoded reporter for diffusion-weighted MRI (Fig. 1a), we used lentiviral transfection to generate CHO, U87 glioblastoma and Neuro 2a neuroblastoma cell lines stably overexpressing this channel, and corresponding control cells expressing a green fluorescent protein (GFP). Expression of AQP1 and GFP were driven by identical constitutive cytomegalovirus (CMV) promoters in U87 and Neuro 2a cells. To implement chemogenetic control of gene expression, we used a doxycycline-regulated CMV promoter in CHO cells engineered to stably express the tetracycline transactivator (rtTA) protein. Pellets of AQP1- and GFP-expressing CHO, U87 glioblastoma and Neuro 2a neuroblastoma cells were then imaged using DWI. A key parameter in diffusion-weighted pulse sequences is the effective diffusion time, Δ_eff_, corresponding to the time interval between dephasing and rephasing gradient pulses^36^,^37^,^45^,^46^,^49^,^59^. Long Δ_eff_ times are important for probing the effects of water exchange between intracellular and extracellular pools because longer times allow a larger proportion of cytoplasmic water molecules to interact with the cell membrane and experience the effects of restriction and exchange^36^,^37^,^48^,^49^,^59^. Correspondingly, Monte Carlo simulations of a packed cellular lattice suggested that the effects of an aquaporin-mediated increase in water diffusion would be most pronounced at Δ_eff_>100 ms (Supplementary Fig. 1). We accessed these longer diffusion times using stimulated echo DWI^46^,^59^,^60^.

Pellets of AQP1-expressing cells appeared much darker in diffusion-weighted images compared with GFP controls for all cell types (Fig. 1b), corresponding to dramatic increases in their apparent diffusion coefficients (ADCs, Fig. 1c). Measured with Δ_eff_=398 ms, AQP1-expressing CHO, U87 and Neuro 2a cells showed 187±12%, 82±5% and 95±3% increases in ADC, respectively, relative to GFP controls (*P*<10^−4^, *n*≥4, *t*-test). The relative increase in ADC was less pronounced using a shorter diffusion time (Δ_eff_=18 ms, Supplementary Fig. 2), consistent with a contrast mechanism based on water exchange across the cell membrane. The larger change in ADC in CHO cells compared with Neuro 2a and U87 is likely to be a consequence of the lower basal ADC in control CHO cells (377.57±20.86 μm^2^ s^−1^ at Δ_eff_=398 ms) compared with control Neuro 2a and U87 cells (539.69±11 and 479.25±21.23 μm^2^ s^−1^, respectively). To establish orthogonality to paramagnetic reporters, we measured the *T*_1_ and *T*_2_ relaxation rates of cells expressing AQP1. Overexpression of this protein did not affect *T*_1_ or *T*_2_ relaxation (Fig. 1d,e, not significant, *P*>0.24, *n*=3, *t*-test), suggesting that AQP1 could be used in combination with genetically encoded *T*_1_ or *T*_2_ contrast agents for multiplexed imaging. Importantly, AQP1 overexpression was non-toxic in all cell lines, as determined using four different assays, including ethidium homodimer staining, measurement of cytosolic ATP content, metabolic activity and lactate dehydrogenase release (Fig. 1f). In addition, no changes in cell morphology were observed under phase contrast microscopy as a result of AQP1 expression (Fig. 1g). We note that we were also able to obtain a significant increase in ADC by transfecting cells with another human aquaporin, AQP4 (Supplementary Fig. 3). However, the percentage increase in ADC for the AQP4-expressing cells (44±6% in CHO cells, *P*=6.2 × 10^−3^, *n*=3, *t*-test) was smaller compared with AQP1. Therefore, we focused on AQP1 for the remainder of this work.

### *AQP1* is a sensitive reporter gene with a large dynamic range

**Figure 2:**
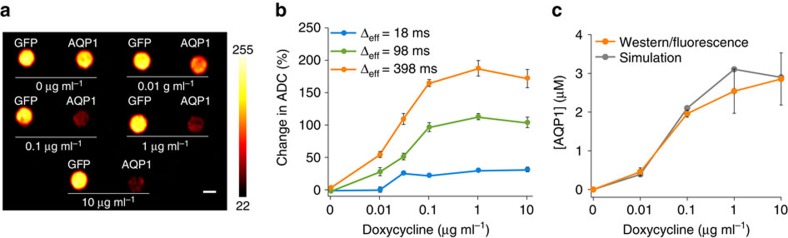
AQP1 reports gene expression over a large dynamic range. (**a**) Diffusion-weighted images (acquired at Δ_eff_=398 ms, *b*=2,089 s mm^−2^) of CHO cells expressing AQP1 or GFP (control) and treated with varying doses of doxycycline to induce transgene expression. Scale bar, 3 mm. (**b**) Percent change in ADC of water in AQP1-expressing CHO cells (relative to control cells expressing GFP) as a function of doxycycline concentration, measured at different diffusion times. *n*=4 biological replicates. Error bars±s.e.m. (**c**) Levels of AQP1 expression on the membrane of CHO cells at different levels of doxycycline induction, estimated based on quantitative western blotting and relative expression of a co-transcribed GFP reporter, compared with values calculated from Monte Carlo simulations based on the ADC results in **b**. *n*≥3 biological replicates. Error bars±s.e.m.

Next, we sought to establish the sensitivity of AQP1 to image varying degrees of gene expression. Our Monte Carlo simulations suggested that ADC values are sensitive to a broad range of cell membrane permeabilities (Supplementary Fig. 1b), providing AQP1 with significant dynamic range. To realize this experimentally, we expressed AQP1 in a dose-dependent manner by supplementing CHO cells with varying concentrations of doxycycline and imaged them with DWI (Fig. 2a,b). The corresponding levels of AQP1 expression were quantified via western blotting and measurements of internal ribosome entry site (IRES)-linked GFP fluorescence (Fig. 2c and Supplementary Fig. 4). A significant increase in ADC was observed across all levels of induction, with differences of 54±5 to 187±12% (*P*≤6.8 × 10^−3^, *n*≥3, *t*-test) compared with controls at doxycycline concentrations of 0.01–1 μg ml^−1^. Notably, the 54% change in contrast corresponds to an estimated AQP1 expression level of just 457±102 nM, consistent with simulation predictions (Fig. 2c). This high sensitivity and large dynamic range will facilitate the use of *AQP1* as a reporter gene in a variety of biomedical applications.

### AQP1 expression is observable in cells in a mixed population

**Figure 3:**
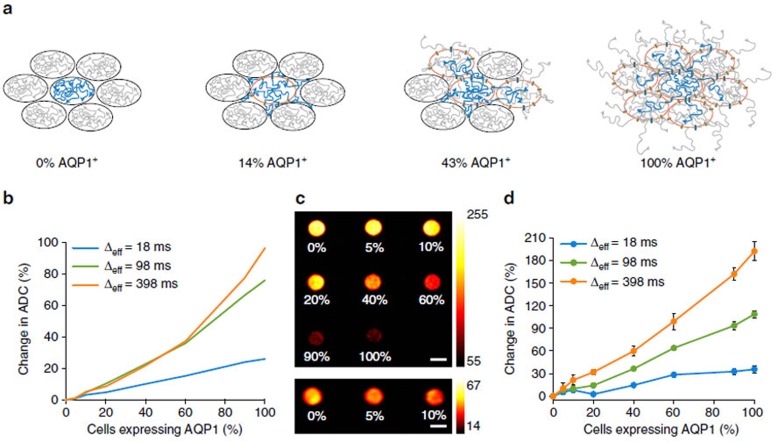
AQP1 expression is observable in mixed cell populations. (**a**) Illustration of the effect of an increasing fraction of AQP1-labelled cells in a tissue on the overall diffusivity of water. (**b**) Monte Carlo simulation predictions of change in ADC as a function of the fraction of cells expressing AQP1 in a mixed cellular lattice. (**c**) Top: diffusion-weighted MRI (acquired at Δ_eff_=198 ms, *b*=2,334 s mm^−2^) of cells comprising AQP1-labelled cells mixed with GFP-labelled control cells in varying proportions. Bottom: image of mixed populations containing 0, 5 and 10% AQP1-expressing cells acquired using Δ_eff_=398 ms, *b*=8,000 s mm^−2^, to maximize contrast for the low AQP1 fraction scenario, smoothed with a Gaussian filter (radius 1.5 pixels). Scale bars, 3 mm. (**d**) Percent change in ADC in mixed AQP1/GFP cell pellets as a function of the fraction of AQP1-expressing cells. *N*=4 biological replicates. Error bars±s.e.m.

The ability to specifically detect small numbers of genetically labelled cells in an otherwise unlabelled population would enable the use of genetically encoded reporters in applications such as *in vivo* tracking of cell-based therapeutics^16^,^61^,^62^. Having shown that AQP1 can appreciably increase water diffusion even at low levels of expression (Fig. 2), we tested whether apparent water diffusion could be significantly increased if AQP1 expression was restricted to a small subset of cells in a mixed population. Intuitively, the relationship between the aquaporin-expressing fraction and water diffusion is expected to be nonlinear, as in small-fraction scenarios cells expressing aquaporin would be surrounded mostly by cells without enhanced water permeability, and the impact of aquaporin expression on overall tissue diffusivity would thereby be diminished (Fig. 3a). However, our Monte Carlo simulations predicted that AQP1-expressing fractions as small as 10% should be sufficient to measurably increase the apparent diffusivity (Fig. 3b and Supplementary Fig. 1c). To verify this experimentally, we imaged mixed populations of AQP1- and GFP-expressing CHO cells in varying proportions (Fig. 3c). Notably, this revealed significant contrast and increase in ADC in cell populations containing just 10% AQP1-expressing cells (21.44±5.21% increase relative to all-GFP controls, *P*=0.03, *n*=4, *t*-test; Fig. 3d). This data suggests that, contrary to initial intuition, diffusional reporter genes such as *AQP1* are suitable for imaging gene expression in heterogeneous or infiltrating cell populations.

### AQP1 enables gene expression imaging in tumour xenografts

**Figure 4:**
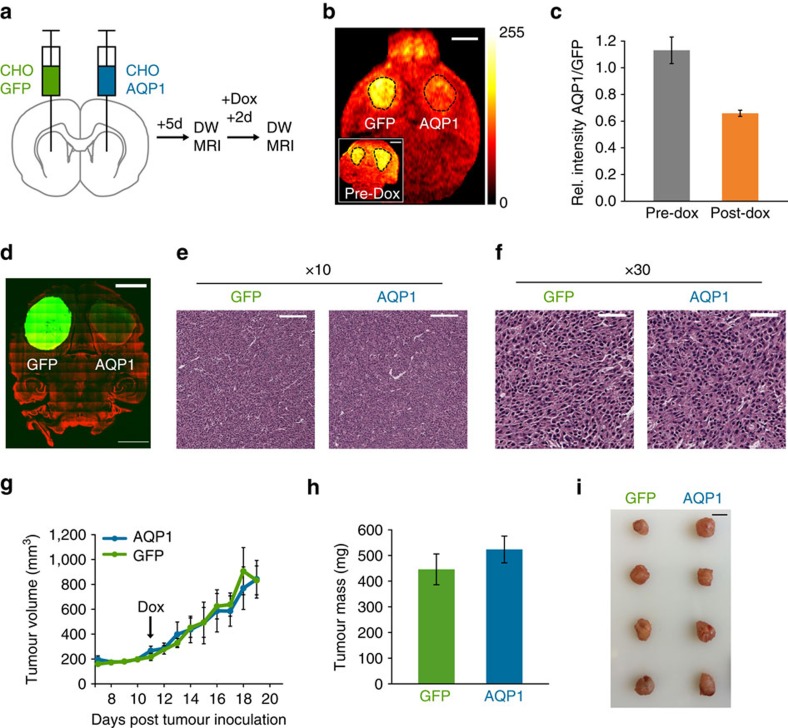
AQP1 enables the imaging of gene expression in intracranial tumour xenografts. (**a**) Experimental approach to establishing bilateral tumours in the striatum, inducing transgene expression, and performing diffusion-weighted MRI. (**b**) Representative diffusion-weighted image of a horizontal brain slice with bilateral tumour xenografts, 48 h after doxycycline injection. Inset shows a diffusion-weighted image of the same mouse acquired before doxycycline injection. Images were acquired at Δ_eff_=98 ms and *b*-value=1,000 s mm^−2^. Dashed lines indicate the tumour ROI(s). Scale bar, 2 mm. (**c**) Average diffusion-weighted image intensity of AQP1-expressing tumours relative to contralateral GFP-expressing tumours before and after doxycycline induction. *n*=5 biological replicates. Error bars±s.e.m. (**d**) Confocal fluorescence image of a representative 100 μm section of a mouse brain implanted with GFP and AQP1 tumours. The AQP1 tumour appears dimmer due to diminished GFP translation from the IRES sequence. Cell nuclei are counterstained using TO-PRO iodide (red). Scale bar, 2 mm. (**e**) Low ( × 10) and (**f**) high ( × 30) magnification images of 5 μm haematoxylin–eosin-stained sections of intracranial tumour xenografts expressing AQP1 and GFP. Scale bars, 30 and 10 μm, respectively. (**g**) Longitudinal measurements of tumour growth in bilateral subcutaneous xenografts induced using doxycycline to express AQP1 or GFP 11 days following tumour inoculation. (**h**) Mean end-point tumour mass and (**i**) images of AQP1- and GFP-expressing subcutaneous tumours harvested 9 days after doxycycline induction of gene expression. *n*=4 biological replicates. Scale bar, 1 cm. Error bars±s.e.m.

To demonstrate the ability of AQP1 to report gene expression *in vivo*, we stereotaxically implanted AQP1 and GFP-transfected CHO cells in the right and left striatum of 5–7-week-old immunodeficient mice. CHO cell xenografts^63^,^64^ were used to enable doxycycline-based regulation of gene expression in the tumours. Tumours were allowed to develop for a period of 5 days, following which we induced transgene expression using intraperitoneal injections of doxycycline. Mice were imaged using diffusion-weighted MRI before and 24–48 h after induction, as outlined in Fig. 4a. As expected, AQP1-expressing tumours are readily distinguishable from contralateral GFP-expressing cells in diffusion-weighted images acquired after induction (Fig. 4b), with the average diffusion-weighted signal intensity in AQP1 tumours decreasing by 39.4±6.5% after doxycycline injection compared with GFP controls (*P*=0.0155, *n*=5, pairwise *t*-test; Fig. 4c and Supplementary Fig. 5). We found that a diffusion time (Δ_eff_) of 98 ms provided the optimal balance of AQP1-dependent contrast and acquisition times for *in vivo* experiments. AQP1 and GFP expression in the bilateral tumours was confirmed by fluorescence imaging of fixed brain tissue slices (Fig. 4d). Haematoxylin–eosin staining revealed no sign of necrosis in either the AQP1- or GFP-expressing tumours, indicating that the change in diffusion-weighted contrast in AQP1 xenografts is caused by AQP1 expression rather than necrosis or other changes in tumour morphology (Fig. 4e,f).

To quantitatively evaluate whether AQP1 overexpression affects tumour growth *in vivo*, we measured growth curves and terminal tumour masses in subcutaneous xenografts established using the same cell lines as the intracranial tumours and induced the same way with doxycycline. The AQP1 and GFP tumours proliferated at similar rates following doxycycline induction and reached statistically indistinguishable end-point masses (Fig. 4g–i, not significant *P*>0.5, *n*=4, pairwise *t*-test). The ability of AQP1 to produce robust induction-dependent MRI contrast in tumour xenografts without affecting tumour growth suggests that this reporter gene could be useful for longitudinal imaging of gene expression *in vivo*.

## Discussion

Our results establish aquaporins, and specifically AQP1, as the first genetically encoded reporter for diffusion-weighted MRI. AQP1-dependent contrast is readily observed in cell cultures, including cells known to have higher levels of endogenous aquaporins (for example, U87 glioblastoma cells^65^) as well as *in vivo* tumour xenografts, and AQP1 expression has no adverse effect on cell proliferation and viability. Aquaporins have several distinct advantages relative to existing MRI reporter genes. First, as a metal-free reporter, aquaporins are not limited by metal bioavailability and do not require the administration of metal ions or chelates. Second, AQP1-dependent contrast can be detected at reasonably low concentrations (∼0.5 μM), which makes it a sensitive MRI reporter gene. Although CEST reporters operating on hyperpolarized xenon can achieve even higher molecular sensitivity^24^,^66^, their use requires elaborate equipment for xenon hyperpolarization and administration, and in the case of gas vesicles, the expression of complex multi-gene clusters. In addition, successful CEST experiments require sophisticated pulse sequences, whereas diffusion-weighted imaging is implemented as a standard technique on clinical MRI scanners. Finally, as a human protein that works without sequence modifications, aquaporin can serve as a fully autologous reporter gene, overcoming concerns about potential immunogenicity faced by xenogeneic and engineered reporters.

AQP1 expression does not affect transverse and longitudinal relaxation rates in cells, which creates the possibility of multiplexed MRI of gene expression by combining aquaporins with existing *T*_1_, *T*_2_ or CEST reporters. In addition, it is conceivable that increased water diffusion in AQP1-expressing cells could enhance the relaxivity of co-localized *T*_1_ or *T*_2_ agents by facilitating paramagnetic relaxation of a larger fraction of water molecules.

One potential limitation of aquaporin as a reporter gene is its negative contrast enhancement, as AQP1 expression results in image darkening in diffusion-weighted MRI. Although negative contrast agents are widely established in MRI, their use warrants a certain degree of caution in light of potential confounding signal dropout from lesions, abscesses and susceptibility artefacts. In the case of aquaporin, confounds from *T*_1_ and *T*_2_ relaxation can be accounted for by mapping ADC rather than acquiring single diffusion-weighted images. To distinguish aquaporin expression from tissue structures with high background diffusivity, such as fluid-filled cysts and necrotic lesions, it may be necessary to combine information from ADC measurements at several Δ_eff_ times and from *T*_1_- and/or *T*_2_-weighted images. Chemogenetic toggling of aquaporin expression, trafficking or degradation could also help identify reporter gene-dependent signals. Finally, although we have demonstrated the application of AQP1 as a non-toxic MRI reporter in three different cell lines and *in vivo* tumours, the broader utility of this reporter gene in the context of different cell types and tissue architectures (for example, epithelial cells and neurons) and alternative biological applications, remains to be investigated in future studies.

Given the ubiquity of DWI and stimulated echo pulse sequences, the imaging of aquaporin-based reporters can be implemented immediately by laboratories with standard MRI equipment. Beyond this, imaging performance could potentially be improved further using alternative pulse sequences specifically designed to produce contrast sensitive to transmembrane water exchange^60^, the further development of which will be stimulated by this work. In addition, we anticipate that the performance of aquaporins as MRI reporters can be further enhanced through molecular engineering of variants with improved or stimulus-gated permeability to enable functional imaging of biologically relevant markers. Overall, the high performance, biocompatibility and engineering capacity of aquaporin reporter genes will enable this remarkably simple new approach to biomolecular MRI to impact many areas of biology and medicine.

## Methods

### Construction of aquaporin and GFP-expressing cell lines

Human *AQP1* (NM_198098.1) and *AQP4* (NM_001650.4) complementary DNAs were ordered from OriGene (Rockville, MD) and subcloned into a lentiviral vector downstream of a constitutive CMV or doxycycline-regulated CMV promoter (Clontech, Mountain View, CA) and an N-terminal FLAG tag. The doxycycline-regulated CMV was used specifically to place AQP1 or GFP under the control of doxycycline using a commercially available CHO cell line (Clontech) that expresses the rtTA transactivator. Enhanced GFP was fused downstream of aquaporin via an IRES sequence. Lentiviral packaging was performed in HEK 293T cells by transfecting 22 μg of packaging plasmid to expresses the capsid genes from a CMV promoter along with 22 μg of insert plasmid harboring the gene of interest (*AQP1-IRES-GFP*, *AQP4-IRES-GFP*, or *GFP*) flanked by long terminal repeat sequences and 4.5 μg of VSV-G plasmid that expresses the vesicular stomatitis virus G protein to enable broad tropism of the lentiviral particles. Transfection was achieved using 25 kDa linear polyethyleneimine (Polysciences, Warrington, PA) at a concentration of 2.58 mg PEI per mg DNA. Approximately 24 h post transfection, the culture medium was supplemented with sodium butyrate at 10 mM concentration, to induce expression of the packaging genes. Virus production was allowed to proceed for 48–60 h following which the virus-laden supernatant was collected, centrifuged at 500 *g* to remove residual HEK 293T cells, mixed with one-tenth the volume of Lenti-X concentrator (Clontech) and incubated at 4 °C for at least 24 h. Lentiviral particles were subsequently sedimented by centrifugation at 1,500 *g* for 45 min at 4 °C and resuspended in 1–2 ml of DMEM medium. Resuspended viral particles were immediately used to transfect CHO, CHO-TetON, Neuro 2a or U87 cells, to generate stable cell lines. For this, the cells were first grown to 70–80% confluency in six-well plates. Spent medium was aspirated from the wells and replaced with 1 ml lentivirus suspension together with 8 μg ml^−1^ polybrene. The cells were spinfected at 2,000 *g* for 90 min at 30 °C, following which the plates were returned to the 37 °C incubator for 48 h to allow gene expression. Control cell lines were generated in the same way to express enhanced GFP from a constitutive or doxycycline-regulated CMV promoter. Cell lines were obtained from American Type Cell Culture Collection (U87, Neuro 2a, HEK 293T, CHO) or from Clontech (CHO-TetON) and used without further validation. Further, we note that none of the cell lines used in the study are listed in the database of cross-contaminated cell lines maintained by the International Cell Line Authentication Committee as of 10/26/2016. Some of the cell lines were periodically checked for Mycoplasma contamination using the MycoAlert detection kit from Lonza.

### Determination of cell viability

Cell viability was determined using four different approaches including staining with ethidium homodimer-1 (Thermo Fisher) and measurement of resazurin reduction (CellTiter-Blue assay, Promega), ATP content (CellTiter-Glo assay, Promega) and lactate dehydrogenase release (CytoOne, Promega). For ethidium homodimer-1 staining, AQP1- and GFP-expressing cells were grown in six-well plates for 48 h, trypsinized and resuspended in 100 μl PBS supplemented with ethidium homodimer-1 at 4 μM final concentration. The cell-dye mixture was allowed to incubate at 4 °C for 1 h in a rotary shaker. Subsequently, 10 μl of the cell suspension was loaded in a disposable hemocytometer (C-chip DHC S02, Incyto) and total number of cells was estimated by imaging the hemocytometer chamber using bright field microscopy. Dead cells stained red and were estimated using fluorescence imaging with a Cy3 filter set. Viability was calculated as the fraction of cells that did not stain using ethidium homodimer-1. For the remaining cytotoxicity assays, AQP1- and GFP-expressing cells were grown in 96-well plates for 24–48 h and treated with the assay reagents as described by the manufacturer. Fluorescence (resazurin reduction and lactate dehydrogenase release) or luminescence (ATP content assay) readouts were measured using a SpectraMax fluorescence plate reader using an excitation wavelength of 560 nm and with the emission filter set to 590 nm for fluorescence, and with an open filter slot with a 1 s integration time for luminescence.

### Quantification of AQP1 expression

AQP1 expression was quantified via western blotting and relative fluorescence measurements. AQP1 expression was induced in CHO cells by treating the cells with doxycycline for 48 h. Membrane fractions were isolated using ProteoExtract native membrane protein extraction kit (EMD Millipore, Billerica, MA) or MEM-PER Plus membrane protein extraction kit (Thermo Fisher) and concentrated ∼30-fold using a 10 kDa centrifugal filter. Alternatively, proteins were concentrated using trichloroacetic acid precipitation (ProteoExtract protein precipitation kit). Proteins were denatured at 37 °C for at least 1 h followed by 95 °C for 5 min and resolved on a denaturing SDS–PAGE gel, transferred to a polyvinylidene difluoride membrane and probed using mouse anti-FLAG primary antibodies ( 0.5 μg ml^−1^ final concentration) and horseradish peroxidase-conjugated goat anti-mouse IgG secondary antibodies (0.4 μg ml^−1^ final concentration). Primary and secondary antibodies were purchased from Sigma (catalogue number F1365) and Santa Cruz Biotech (catalogue number sc-2005). Signal detection was achieved using the Clarity chemiluminescent substrate (Biorad, Hercules, CA) using an exposure time of 1–10 s. AQP1 expression was quantified from a calibration curve of known quantities (100 to 400 ng) of FLAG-tagged bacterial alkaline phosphatase (Sigma Aldrich, St Louis, MO) that was simultaneously loaded, stained and imaged on the same blot (Supplementary Fig. 4a). As AQP1 expression in cells induced with 0.01 μg ml^−1^ doxycycline was below the chemiluminescence detection limit of our western blotting, we estimated AQP1 concentration in this case by quantifying doxycycline dependent fluorescence of IRES-linked GFP. In particular, we measured GFP fluorescence in cells induced using various concentrations of doxycycline to derive a dose-response curve for transcriptional regulation by doxycycline. Based on this, we estimated a relative response ratio of 0.18±0.03 (*n*=4) between GFP expression in low (0.01 μg ml^−1^) and high (1 μg ml^−1^) doxycycline conditions. As *AQP1* and *GFP* are co-transcribed into a single polycistronic construct, we expect the doxycycline dose-response curve to be conserved for the *AQP1* messenger RNA as well. This enabled us to extrapolate the concentration of AQP1 in the low doxycycline scenario by multiplying the measured AQP1 concentration at high doxycycline induction (2.54±0.46 μM based on western blotting, *n*=5) by the response ratio of 0.18. For the fluorescence measurements, doxycycline-treated cells were lysed using RIPA buffer and GFP fluorescence was measured in the cell lysates using a SpectraMax fluorescence plate reader with excitation wavelength set to 450 nm. Fluorescence emission was quantified by integrating the emission spectrum between 480 and 610 nm. Before fluorescence measurements, lysate concentrations were adjusted, to ensure equal total protein levels across samples.

### Diffusion-weighted MRI of cell pellets

For diffusion-weighted MRI, cells were grown for 48 h, trypsinized, resuspended in 100 μl PBS and centrifuged at 500 *g* for 5 min in 0.2 ml PCR tubes, to produce a compact pellet. Subsequently, the tubes were loaded in wells molded in a 1% agarose phantom and imaged using a Bruker 7T horizontal bore MRI scanner equipped with a 7.2 cm diameter bore transceiver coil for radio frequency excitation and detection. Diffusion-weighted images were acquired on a 1.5 or 2 mm-thick horizontal slice through the cell pellets using a stimulated echo DWI sequence with the following parameters: echo time, *T*_E_=24.5 ms, repetition time, *T*_R_=2 s, number of excitations=1–3, gradient duration, *δ*=7 ms, matrix size=256 × 256, field of view (FOV)=3.5 × 6.5 cm^2^. The gradient interval (Δ) was varied from 20 to 400 ms to generate effective diffusion times (Δ_eff_=Δ−*δ*/3) of 18–398 ms in each experiment. Single-axis diffusion gradients were applied and gradient strength was varied to generate *b*-values in the range 0–800 s mm^−2^. For each value of Δ_eff_, ADC was calculated from the slope of the logarithmic decay in MRI signal intensity versus b-value. Images were analysed using custom macros in ImageJ (NIH). A linear 8-bit colour scale were used to facilitate the visualization of the relevant contrast in each figure. Least-squares regression fitting was performed using Origin 2016 or Matlab version 9 (2016).

### *T*_1_- and *T*_2_-weighted MRI of cell pellets

*T*_1_-weighted images were acquired using a rapid acquisition with relaxation enhancement sequence with the following parameters: *T*_E_=9.6 ms, rapid acquisition with relaxation enhancement factor=4, *N*_EX_=2, matrix size=128 × 256, FOV=8 × 5 cm^2^, slice thickness=1.5 mm and receiver bandwidth=50,505.1 Hz. Variable *T*_R_ times were used including 146.19, 321.47, 519.98, 748.83, 1018.9, 1348.72, 1771.99, 2363.81, 3355.44 and 7500, ms. *T*_1_-values were estimated from the following equation:


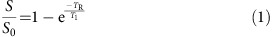


where *S*_0_ is the equlibrium magnetization. *T*_2_-weighted images were acquired using a Car–Purcell–Meiboom–Gill pulse sequence with the following parameters: *T*_E_=11 ms, *T*_R_=1.5 s, number of echoes=63, number of excitations=4, matrix size=256 × 256, FOV=8 × 5 cm^2^, slice thickness=1.5 mm and receiver bandwidth=50,505.1 Hz. *T*_2_ relaxation rates were estimated by fitting the first 19 echoes to the signal decay equation:


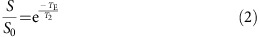


All images were analysed using custom macros in ImageJ (NIH) and least-squares regression fitting was performed using OriginLab. We report average *T*_1_ and *T*_2_ measurements for *n*=4.

### Mouse xenograft model

To prepare cells for intracranial tumour implantation, AQP1- and GFP-expressing CHO-TetON cells were grown for 48 h, trypsinized, centrifuged at 500 *g* for 10 min and resuspended in 100 μl serum-free DMEM. Female NOD/SCID/γ-mice between 5 and 7 weeks of age (Jackson Laboratory, Bar Harbor, ME) were anaesthetized with 2.5% isoflurane and 10^5^ AQP1-expressing CHO cells were injected stereotaxically into the right striatum. Coordinates of the injection sites with respect to bregma were as follows: 1 mm anterior, 2 mm lateral and 1–3 mm ventral from the surface of the calvaria. The same number of control GFP-expressing CHO cells were implanted in the left striatum of the same animal.

For longitudinal measurements of tumour volume, subcutaneous xenografts were established by injecting 3 × 10^6^ AQP1 and GFP CHO cells (prepared as described above and resuspended in Matrigel) into the right and left hind limbs of female NOD/SCID/γ-mice. Gene expression was induced by intraperitoneal injection of 75 μg doxycycline 11 days following tumour inoculation. Tumour size was measured daily using callipers and tumour volume was calculated as 0.52 × (short axis)^2^ × (long axis). A sample size of *n*=4 biological replicates was deemed adequate for a power (expressed as 1−*β*) of 0.80, calculated based on the difference in AQP1 and GFP groups observed *in vitro*. In addition, the tumour models are well established and tumour growth is stable, which obviated the need for a larger sample size. No surviving animals were excluded from the final analysis. Tumour inoculation sites were not randomized and investigators were not blinded to the experiments. All animal experiments were approved by the Institutional Animal Care and Use Committee of the California Institute of Technology.

### Diffusion-weighted MRI of brain tumour xenografts

Diffusion-weighted imaging of mouse xenografts was performed using a Bruker 7T horizontal bore MRI scanner. Radio frequency excitation was delivered by a 7.2 cm diameter bore volume coil and detection was achieved using a 3 cm diameter surface coil. Mice were anaesthetized using 1–2% isoflurane. Respiration and temperature were continuously monitored using a pressure transducer (Biopac Systems) and fibre optic rectal thermometer (Neoptix). Warm air was circulated to maintain body temperature at 30 °C. Tumour formation was confirmed by acquiring diffusion-weighted images 5 days following xenograft implantation, after which mice were intraperitoneally injected with 75 μg doxycycline to induce expression of AQP1 and GFP in the tumours. A second set of diffusion-weighted images was acquired 24–48 h following doxycycline injection. Preliminary diffusion-weighted images to locate the tumours were first acquired on horizontal slices using a three-dimensional echo planar imaging stimulated echo DWI sequence with the following parameters: *T*_R_=2.5 or 3 s, *T*_E_=25.7 ms, *δ*=7 ms, Δ=100 ms, *b*=1,000 s mm^−2^, number of excitations=9, matrix size=16 × 128 × 128 and FOV=1.59 × 1.29 × 0.74 cm^3^. After identifying an appropriate tumour-bearing slice, two-dimensional echo planar imaging diffusion-weighted images were acquired at the slice using similar parameters but with a slice thickness of 1–2 mm, *T*_R_=5 s, number of excitations=144–256.

### Histological analyses of brain tissue

Mouse tumours were evaluated for gene expression and signs of necrosis via fluorescence imaging of 100 μm-thick histological sections and haematoxylin–eosin staining of 5 μm-thick paraffin-embedded sections. For histological analyses, mice were anaesthetized by intraperitoneal injection of ketamine (100 mg kg^−1^ of body weight) and xylazine (10 mg kg^−1^ of body weight), and transcardially perfused first with PBS containing heparin (10 units ml^−1^, Sigma Aldrich) and subsequently with 4% w/v paraformaldehyde (Sigma Aldrich). Following perfusion, the brain was harvested and fixed in 4% w/v paraformaldehyde for 2 h at room temperature and washed three times with PBS. Axial brain sections of 100 μm-thickness were obtained using a vibratome (Leica Biosystems, Buffalo Grove, IL). Free-floating sections were incubated for 30 min at room temperature with a 1 μM solution of TO-PRO-3 Iodide nuclear stain in PBS (Thermo Fisher Scientific, Waltham, MA). Stained sections were washed three times with PBS and mounted on glass slides with ProLong Diamond Antifade Mountant (Thermo Fisher Scientific) and imaged using a confocal microscope with GFP- and Cy5-specific filter sets. Haematoxylin–eosin staining was performed by the Translational Pathology Core Laboratory in the University of California, Los Angeles.

### Monte Carlo simulations of water diffusion in cells

We developed a model for restricted water diffusion and exchange in cells, building on the previously described Karger and Szafer models of tissue water diffusion^48^,^67^,^68^. We modelled cell pellets as a face-centered cubic lattice packed with 108 spherical cells (Supplementary Fig. 1a) with water molecules distributed randomly throughout the lattice at *t*=0. Cell radii were sampled from a normal distribution with a mean of 6.8 μm and a s.d. of 1.2 μm. We set the simulation time step *τ*=*50* μs and at each time step, water molecules were propagated in a three-dimensional random walk with step size given by 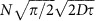 in each direction. Here, *N* is sampled from a random normal distribution and *D* is the free diffusion coefficient of water at 12.9 °C (the bore temperature of our MRI scanner) in the intracellular compartment (554.7 μm^2^ s^−1^) or in the extracellular space (1664.2 μm^2^ s^−1^)^69^. If a water molecule encounters a membrane, the propagation step is recalculated and the molecule either transmitted or reflected off the membrane with a probability given by


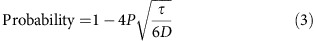


wherein *P* is the membrane permeability and *D* is the free diffusion coefficient of water in the intracellular compartment. Diffusion paths were simulated in Python and the ADC was calculated using Matlab as described in the Szafer model^48^:


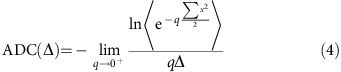


where Σ*x*^2^ represents the sum square displacement of a water molecule from its starting position and *q* is given by (*γδg*)^2^, where *γ* is the gyromagnetic ratio, *g* is the gradient strength and *δ* is the duration of the pulsed diffusion gradient. We note that *b*-value is calculated as:


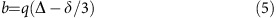


In the first set of simulations (ADC versus permeability), we varied the cell permeability from 0.034 to 0.39 μm ms^−1^ and calculated ADC(Δ) for each value of cell permeability. In the second set of simulations (ADC versus fraction of AQP1-expressing cells), the permeability of AQP1-expressing cells and control cells were fixed at 0.14 and 0.039 μm ms^−1^ respectively, in accordance with previously published values^55^. We incrementally varied the fraction of cells expressing AQP1 and for each composition, simulated 3 × 10^4^ (nonunique) random arrangements of AQP1-expressing and control cells to exclude geometry or arrangement dependent bias in the results. ADC(Δ) was estimated corresponding to varying fractions of AQP1-expressing cells in the population.

### Estimation of AQP1 expression from Monte Carlo simulations

Based on the simulated trend of ADC as a function of cell permeability, we calculated permeability values of CHO cells induced with various concentrations of doxycycline and for which ADC values had been experimentally measured. Permeability values calculated in this manner ranged from 0.074 to 0.55 μm ms^−1^ corresponding to 0.01 and 1 μg ml^−1^ doxycycline concentrations. Next, permeability values were converted to volumetric flow rates by taking their product with the average surface area of a CHO cell (380 μm^2^). AQP1 concentration was estimated based on the previously reported unit channel conductance of 6 × 10^−5^ μm^3^ ms^−1^ (refs 53, 55).

### Statistical analysis

For statistical significance testing, we used two-sided homoscedastic *t*-tests with a significance level of type I error set at 0.05 for rejecting the null hypothesis. Paired-sample *t*-tests were used where indicated. Homogeneity of variances between data sets was verified using Bartlett's test or *F*-test, although we note that the statistical significance of our results remains conserved on using the Welch's *t*-test for heteroscedastic distributions. Normal distribution of data sets was verified using the Kolmogorov–Smirnov test with a significance level of 0.01.

### Data availability

All data presented in support of the findings in this study and plasmids are available from the authors upon request.

### Code availability

Python and MATLAB scripts for Monte Carlo simulations are available at http://shapirolab.caltech.edu/?page_id=525.

## Additional information

**How to cite this article:** Mukherjee, A. *et al*. Non-invasive imaging using reporter genes altering cellular water permeability. *Nat. Commun.* **7,** 13891 doi: 10.1038/ncomms13891 (2016).

**Publisher's note:** Springer Nature remains neutral with regard to jurisdictional claims in published maps and institutional affiliations.

## Supplementary Material

Supplementary Information Supplementary Figures
