# Supplementary material for: Non-invasive imaging using reporter genes altering cellular water permeability
Source: Nat Commun. 2016 Dec 23;7:13891. doi: 10.1038/ncomms13891 (PMC5196229; doi:10.1038/ncomms13891)
Supplement: Supplementary Information — Supplementary Figures [file ncomms13891-s1.pdf]

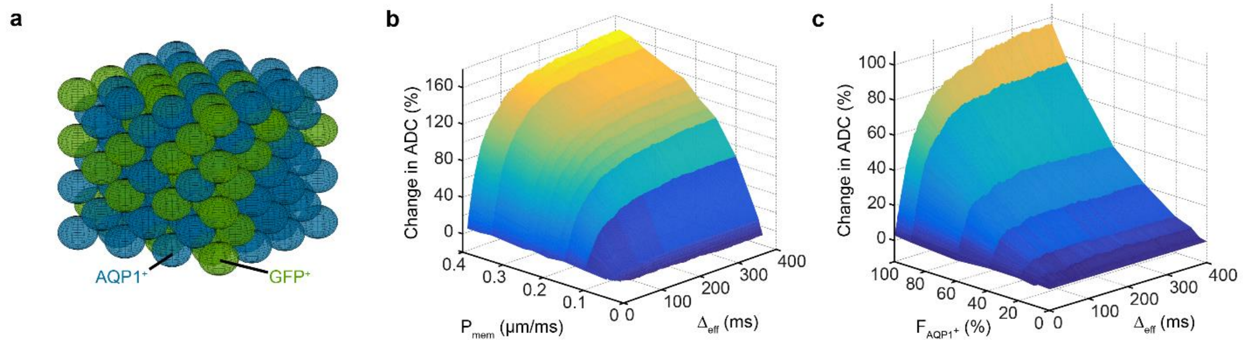

Supplementary Figure 1. Monte Carlo simulations of water diffusion in $AQP1^+$ and GFP^+ (control) cells as a function of cell membrane permeability (P_{mem}), effective diffusion time (Δ_{eff}), and percentage of AQP1-labeled cells (F_{AQP1+}). (**a**) Mixed populations of $AQP1^+$ and GFP^+ cells were modeled by randomly distributing $AQP1^+$ and GFP^+ cells in the lattice to simulate 3 x 10^4 (nonunique) random arrangements of heterogeneous cell populations corresponding to varying fractions of $AQP1^+$ cells. (**b**) ADC increases with increasing cell permeability in a homogeneous cell population, with the percent change in the ADC (measured relative to control cells with a basal permeability of 0.035 µm ms^{-1}) being most pronounced at longer diffusion times. (**c**) ADC increases in a nonlinear fashion with increasing fractions of AQP1-labeled cells in a mixed population comprising $AQP1^+$ and GFP^+ cells.

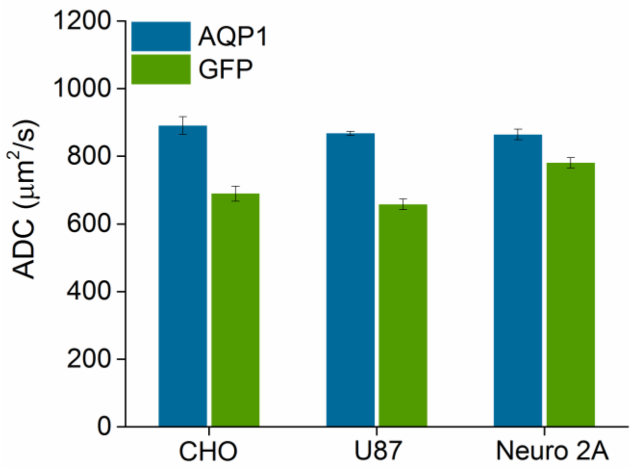

Supplementary Figure 2. Apparent diffusion coefficient of water in AQP1 and GFP-expressing CHO cells measured at short diffusion times (Δ_{eff} = 18 ms). Percent change in ADC on account of AQP1 expression is smaller at short diffusion times. Error bars represent standard error of mean (SEM) for n=4 biological replicates.

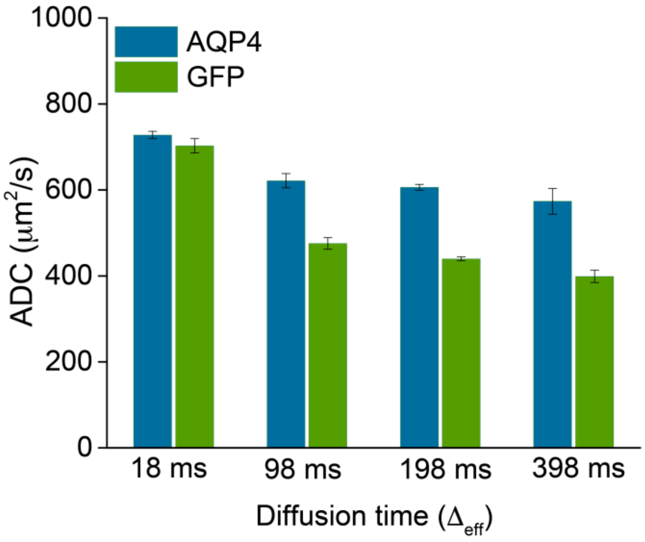

Supplementary Figure 3. AQP4 is a genetically encoded reporter for diffusion weighted MRI. AQP4 expression enhances water diffusion in CHO cells relative to GFP controls, albeit not to the same extent as AQP1. Error bars represent standard error of mean (SEM) for n=4 biological replicates.

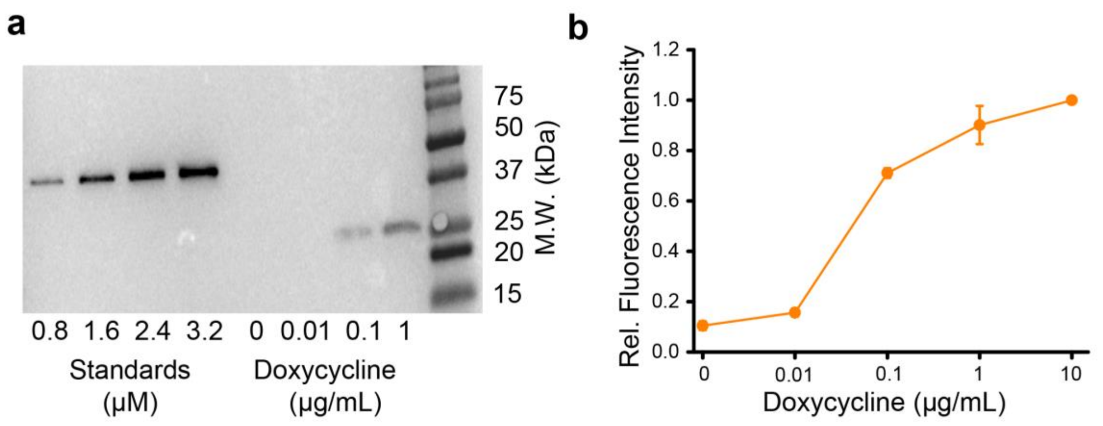

Supplementary Figure 4. Western blotting of AQP1 expressed on the membrane of CHO cells. (**a**) Representative western blot of FLAG-tagged bacterial alkaline phosphatase standards (indicated in terms of their equivalent cellular concentration) alongside lysate fractions from CHO cells induced with the indicated concentrations of doxycycline. (**b**) GFP fluorescence from CHO cells induced with the indicated concentrations of doxycycline. The fluorescence comes from IRES-GFP downstream of AQP1. Error bars represent standard error of mean (SEM) for n=4 biological replicates.

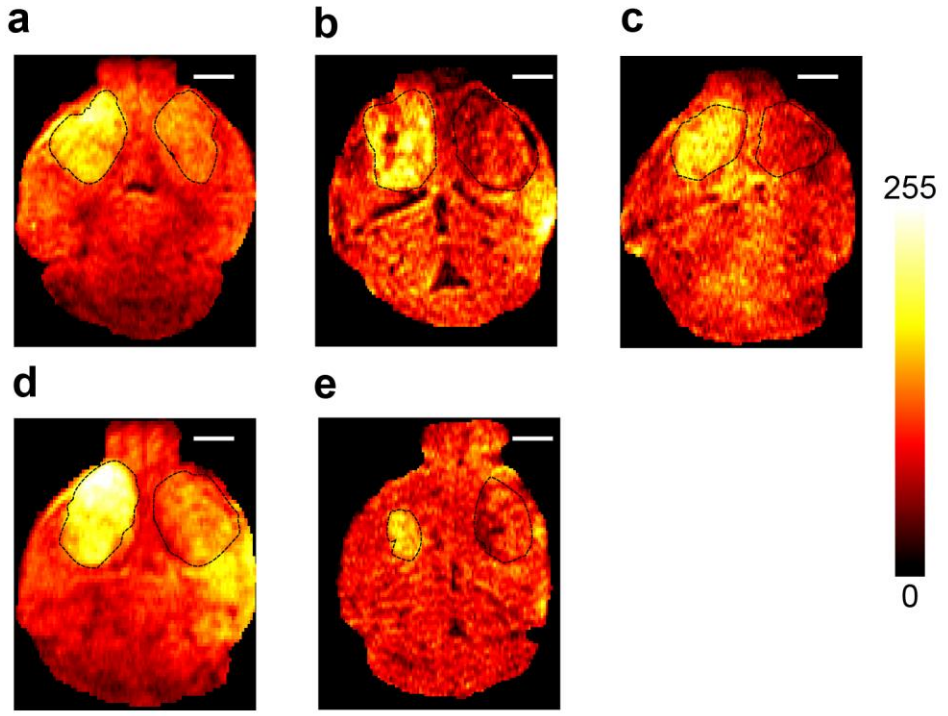

Supplementary Figure 5. Diffusion weighted images of horizontal slices of the mouse brains with bilateral tumor xenografts, acquired 48 hours following intraperitoneal injection of doxycycline. AQP1-expressing tumors (right striatum) are visibly dimmer than the contralateral GFP-expressing tumors (left striatum). Diffusion weighted images were acquired using a DWI sequence with Δ_{eff} = 98 ms and b = 1000 s mm^{-2} using a Bruker 7T horizontal bore MRI. Tumor ROI(s) are indicated using solid black lines. Scale bar is 2 mm.
